# Supplementary material for: Identification of photoperiod-induced specific miRNAs in the adrenal glands of Sunite sheep (Ovis aries)
Source: Front Vet Sci. 2022 Jul 22;9:888207. doi: 10.3389/fvets.2022.888207 (PMC9354845; doi:10.3389/fvets.2022.888207)
Supplement: Supplementary file 1 [file Data_Sheet_1.ZIP › Supplementary file/miRDeep2 file.zip!/miRDeep2 file/Novel_Structure/SP42A2/Novel_349.pdf]

Provisional ID	:	NC_019462.2_15102
Score total	:	7.7
Score for star read(s)	:	3.9
Score for read counts	:	0.1
Score for mfe	:	2.1
Score for randfold	:	1.6
Score for cons. seed	:	
Total read count	:	12
Mature read count	:	11
Loop read count	:	0
Star read count	:	1

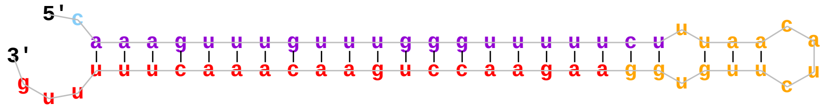

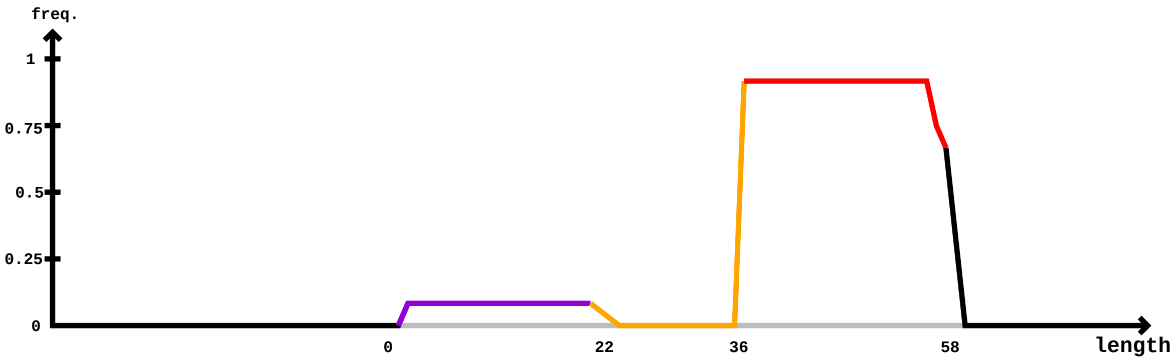


```
                                  Star                             Mature
5'- uguaauuuaauggaauauuauauuggguuggccacaaaguuuguuuggguuuuucuuuaacaucuugugggaagaaccugaacaaacuuuuugggcaaccccauauaaaacaa   -3'   obs
    uguaauuuaauggaauauuauauuggguuggccacaaaguuuguuuggguuuuucuuuaacaucuugugggaagaaccugaacaaacuuuuugggcaaccccauauaaaacaa         exp
    .................((((((.((((((.(((.(((((((((((((((((((((.(((....))).))))))))))))))))))))).))).)))))).)))))).....   reads   mm   sample
    ...................................aaaguuuguuuggguuuuucu........................................................   1       0    seq
    ......................................................................aaAaaccugaacaaacuuuu......................   2       1    seq
    ......................................................................aaAaaccugaacaaacuuuuu.....................   1       1    seq
    ......................................................................aaAaaccugaacaaacuuuuug....................   7       1    seq
    ......................................................................aagaaccugaacaaacuuuuug....................   1       0    seq
```
